# FESTIval: A versatile framework for conducting experimental evaluations of spatial indices

**DOI:** 10.1016/j.mex.2019.10.006

**Published:** 2019-10-16

**Authors:** Anderson C. Carniel, Ricardo R. Ciferri, Cristina D.A. Ciferri

**Affiliations:** a Federal University of Technology – Paraná, Dois Vizinhos, PR 85660-000, Brazil; b Department of Computer Science, University of São Paulo, São Carlos, SP 13566-590, Brazil; c Department of Computing, Federal University of São Carlos, São Carlos, SP 13565-905, Brazil

**Keywords:** Spatial indexing, Spatial access methods, Benchmark, Disk-based spatial index, Flash-aware spatial index, Flash memory, R-trees, eFIND

## Abstract

The use of a spatial index is a common strategy to improve the performance of spatial queries in spatial database systems and Geographic Information Systems. Choosing the right spatial index to be employed in a given context requires a quantitative method to analyze the performance of spatial indices. This is done through extensive experimental evaluations. However, conducting these evaluations is an expensive, error-prone, and challenging task because (i) spatial objects are complex data to manage, (ii) spatial indices can apply different parameter values and thus assume distinct configurations, and (iii) there are indices specifically developed for different storage systems, such as disks and flash memories. In this article, we propose FESTIval, a versatile framework for conducting experimental evaluations of spatial indices. FESTIval has the following main advantages:

• the support for different types of disk-based and flash-aware spatial indices;
• the specification and execution of user-defined workloads;
• the use of a data schema that stores index configurations and statistical data of executed workloads.

Because of its characteristics, FESTIval allows users to reproduce executed experiments. Further, FESTIval provides an extensible environment, where any spatial dataset can be handled by spatial indices. FESTIval has been used to validate new proposals of flash-aware spatial indices, such as eFIND-based indices.

## Graphical abstract

**Figure:**
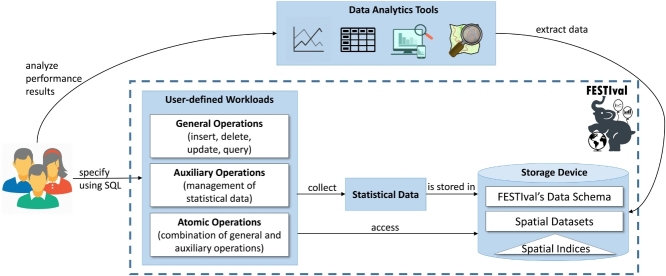

## Method details

This article introduces the *Framework to Evaluate SpaTial Indices* (*FESTIval*), a versatile method for conducting experimental evaluations of spatial indices. Before describing the details of FESTIval, we shortly discuss the context and motivation behind its development.

### Context and motivation

*Spatial database systems* and *Geographic Information Systems* (GIS) widely make use of spatial indices to accelerate the processing of spatial queries, such as *spatial selections*, *range queries*, and *point queries* [1], [2]. A huge set of spatial indices has been proposed in the literature. In general, a spatial index groups nearest spatial objects in index pages. Commonly, these index pages are nodes in hierarchical structures. This organization allows us to avoid the processing of data where the answer of spatial queries certainly cannot be found.

Many spatial indices are designed for manipulating spatial objects stored in magnetic disks like *Hard Disk Drives* (HDDs). Hence, these indices, termed *disk-based spatial indices*, deal with the slow mechanical access and rotational delay of HDDs. Examples of traditional disk-based spatial indices are the R-tree [3] and its variants, the R*-tree [4] and the Hilbert R-tree [5]. The R-tree is able to index spatial objects of any type (e.g., point, line, and region) by employing their minimum bounding rectangles (MBRs) organized in a hierarchical form. The R*-tree improves the insertion algorithm of the R-tree by employing a set of criteria for organizing the entries among the nodes of the tree. The Hilbert R-tree combines the Hilbert curve with the R-tree by using the Hilbert values of the nodes’ entries; thus, the Hilbert R-tree employs the searching algorithm of the R-tree and an insertion algorithm very similar to the insertion algorithm of the B-tree [6]. These spatial indices are surveyed in [1].

The development of spatial indices for newer storage devices like *flash-based Solid State Drives* (SSDs) has attracted the attention of the research community [7], [8], [9], [10], [11]. The main reason is that SSDs have several improved characteristics than HDDs, such as smaller size, lighter weight, lower power consumption, and faster reads and writes. However, SSDs have intrinsic characteristics that introduce several system implications [12], [13], [14], [15], such as the asymmetric costs between reads and writes, the performance interference of interleaved reads and writes, and the read disturbance management.

To take into account the intrinsic characteristics of SSDs, *flash-aware spatial indices* have been proposed in the literature, such as FAST-based indices [16], the FOR-tree [17], and eFIND-based indices [18], [19]. While the FOR-tree ports the R-tree to SSDs, FAST and eFIND are generic approaches to porting any type of hierarchical index to SSDs. A common focus of these approaches is on decreasing the number of random writes to the SSD by employing an in-memory buffer to store index modifications. If this buffer is full, a flushing algorithm is executed. This operation may deploy a flushing policy to pick some index modifications stored in the buffer to be sequentially written to the SSD. eFIND-based indices distinguish themselves because eFIND is based on a distinct set of design goals that exploits the positive characteristics of SSDs. For instance, eFIND has specific data structures and algorithms to mitigate the effects of reads on frequent locations and interleaved reads and writes.

With the increasing number of spatial indices, choosing the best spatial index to be employed in a given context requires the execution of extensive performance evaluations [1]. However, conducting these evaluations is an expensive, error-prone, and challenging task because (i) spatial objects are complex data to manage, (ii) spatial indices can apply different parameter values and thus assume distinct configurations, and (iii) there are indices specifically developed for different storage systems, such as disks and flash memories. A performance evaluation usually requires the execution of *user-defined workloads* on a given spatial dataset. A workload consists of a set of index operations, such as insertions, deletions, or updates of spatial objects, and the processing of spatial queries.

To the best of our knowledge, there are no methods that provide needed functionalities for creating and executing workloads to benchmark disk-based and flash-aware spatial indices on different storage devices. The reason is that existing approaches [20], [21], [22], [23] face several problems (see next section). In general, they are not extensible since users are not able to define their own workloads. More importantly, they do not provide support for flash-aware spatial indices.

In this article, we extend our previous work [39] by proposin FESTIval, a versatile framework for conducting experimental evaluations of spatial indices that:

• provides support for different types of disk-based and flash-aware spatial indices;
• allows the specification and execution of user-defined workloads under a unique environment;
• allows the reproduction of executed experiments;
• employs a data schema that stores index configurations and statistical data of executed workloads.

### Related work

Benchmarking spatial indexing structures in spatial database systems and GIS helps users to identify the best spatial indices for their applications. There are a few approaches available in the literature [20], [21], [22], [23] that provide tools for conducting experimental evaluations of spatial indices. AMDB [20] and BASIS [21] are approaches that enable the performance analysis of different spatial indices like the R-tree and the R*-tree under the same environment. However, they do not allow the definition of user-defined workloads. In addition, they do not provide support for flash-aware spatial indices.

LOCUS [22] is a benchmark focused on conducting performance evaluations of *Location-Based Services*, which consider point datasets representing mobile users. It specifies a set of workloads for executing index operations considering different types of spatial queries. Unfortunately, LOCUS does not provide support for other spatial data types (e.g., complex regions) and face the same problems of the aforementioned approaches.

The tool employed in [23] provides a unique environment to conduct experimental evaluations of different spatial indices. This tool permits users to implement their workloads by using C/C++ language. However, its focus is on in-memory spatial databases; thus, it assumes that the whole dataset fits in the main memory without accesses to external storage devices like HDDs and SSDs.

Researchers from the spatial database and GIS communities also often define their own datasets and workloads in their experiments (e.g., in [3], [4], [5], [7], [16], [17]). The main problem of conducting such standalone experiments is the extra effort with implementations since none of the aforementioned approaches offers a versatile environment for (i) defining new workloads, (ii) implementing other spatial indices, (iii) varying parameter values, and (iv) storing the statistical data of executed workloads. Further, reproducing or extending the standalone experiments can be a problematic task because the employed implementations and datasets are possibly not publicly available.

On the other hand, in this article we solve the aforementioned problems by proposing FESTIval. FESTIval distinguishes itself because it allows the definition of user-defined workloads in a unique and common environment. Further, it allows the specification and execution of different configurations of disk-based and flash-aware spatial indices. Statistical data of these executions are stored in an integrated data schema. By posing queries on this data schema, users are able to retrieve and analyze performance results.

### FESTIval

FESTIval is an open-source PostgreSQL extension implemented in C by using the extensibility provided by the PostgreSQL internal library.^1^ FESTIval is also based on the PostGIS,^2^ a widely used PostgreSQL extension to manage spatial objects. To process topological relationships in spatial queries, FESTIval further requires GEOS,^3^ an open-source geometry engine for computing spatial predicate functions and spatial operators. The complete documentation of FESTIval is available at https://accarniel.github.io/FESTIval/.

Currently, FESTIval provides support for the following disk-based spatial indices: the R-tree, the R*-tree, and the Hilbert R-tree. FESTIval also provides support for the following flash-aware spatial indices: the FAST R-tree, the FAST R*-tree, the FAST Hilbert R-tree, the FOR-tree, the eFIND R-tree, the eFIND R*-tree, and the eFIND Hilbert R-tree. Further, FESTIval allows the execution of user-defined workloads on real storage devices (e.g., HDDs and SSDs) and on the Flash-DBSim [24], which is a flash simulator that emulates the behavior of flash memory in the main memory. The use of a flash simulator is useful because the Flash Translation Layer (FTL) [25] of a real flash memory usually does not provide access to the number of internal operations actually performed on the flash memory.

**Fig. 1:**
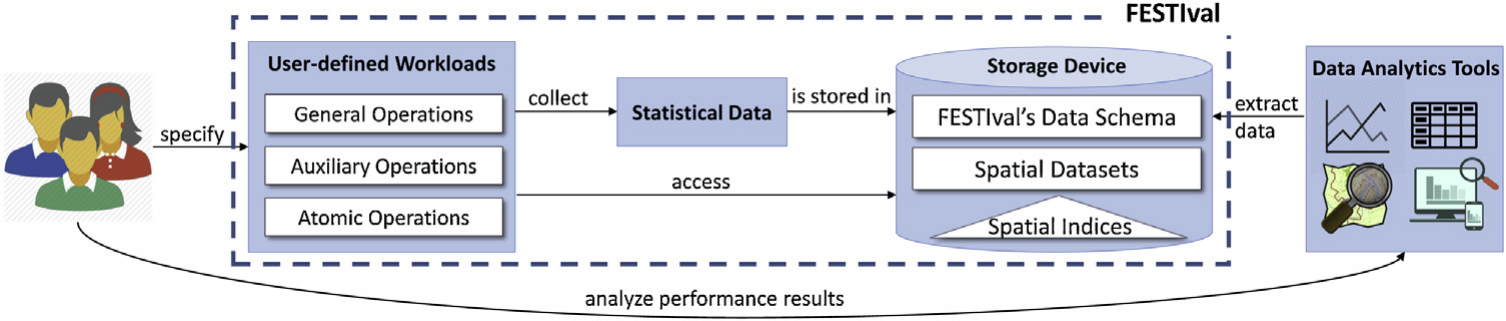
The overview of FESTIval.

Fig. 1 depicts a general view of FESTIval, which is detailed as follows.

*Workloads*. FESTIval allows the creation and execution of user-defined workloads by using the Structured Query Language (SQL). By using the FESTIval's SQL functions, a user (e.g., database administrator, researcher, and software developer) is able to define the sequence of index operations that should be executed and then analyzed. Index operations include insertions, updates, and deletions of spatial objects, and the execution of spatial queries (i.e., *general and atomic operations*). Further, users can also determine the exact moment that statistical data should be collected and stored in the FESTIval's data schema (i.e., *auxiliary operations*).

*Storage device*. It stores three main elements: (i) the spatial indices, (ii) the spatial datasets, and (iii) the FESTIval's data schema. The spatial indices are handled by FESTIval during the execution of workloads, whereas the spatial datasets provide spatial objects to these indices. The FESTIval's data schema stores information of spatial datasets, parameters used by spatial indices, and statistical data of executed workloads. Collected statistical data can be used in mathematical models to measure the performance of spatial indices, considering employed parameter values, characteristics of the spatial dataset, and the employed storage device.

#### The FESTIval's data schema

**Fig. 2:**
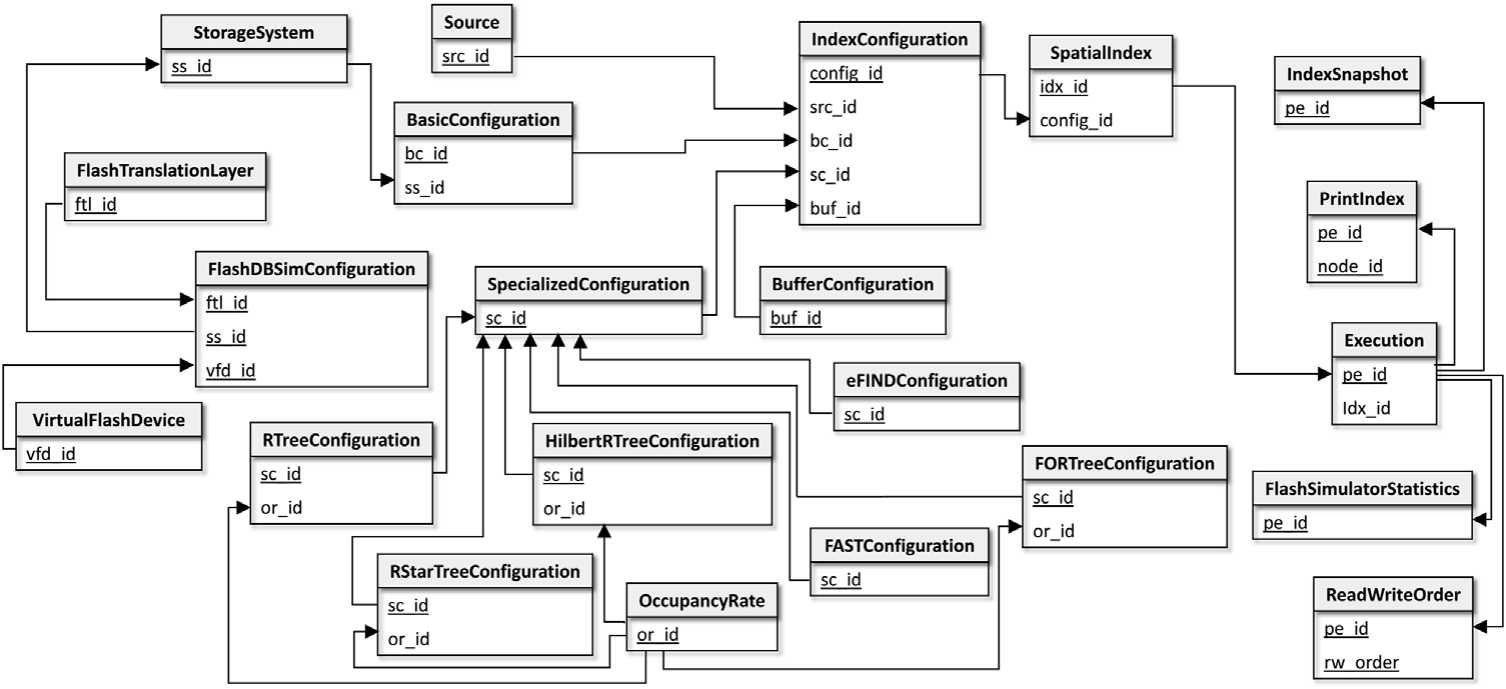
The FESTIval's logical data schema. This figure shows only the primary and foreign keys of the relational tables to illustrate the relationships among the tables. Primary keys are highlighted. The relationship between a primary key and a foreign key is represented by a directed arrow from the primary key to the foreign key. Table 1 details the attributes of each relational table of this figure.

Fig. 2 depicts the FESTIval's logical data schema, called *fds*. In this figure, we only show the primary and foreign keys of the relational tables to illustrate their relationships. Table 1 enriches this figure by listing the attributes of each relational table. Here, we only provide a general view of this schema, detailing the most important tables only. The complete description can be found at the FESTIval's documentation.

**Table 1:** Attributes of each relational table of the FESTIval's data schema.

Relational table	Attributes
Source	src_id, schema_name, table_name, column_name, pk_name
BasicConfiguration	bc_id, ss_id, page_size, io_access, refinement_type
StorageSystem	ss_id, storage_system, description
FlashDBSimConfiguration	ss_id, ftl_id, vfd_id
VirtualFlashDevice	vfd_id, nand_device_type, block_count, page_count_per_block, page_size1, page_size2, erase_limitation, read_random_time, read_serial_time, program_time, erase_time
FlashTranslationLayer	ftl_id, ftl_type, map_list_size, wear_leveling_threshold
BufferConfiguration	buf_id, buf_type, buf_size
SpecializedConfiguration	sc_id, description
RTreeConfiguration	sc_id, or_id, split_type
RStarTreeConfiguration	sc_id, or_id, reinsertion_perc_internal_nodes, reinsertion_perc_leaf_nodes, reinsertion_type, max_neighbors_exam
HilbertRTreeConfiguration	sc_id, or_id, order_splitting_policy
FORTreeConfiguration	sc_id, or_id, buffer_size, flushing_unit_size, ration_flushing, x, y
FASTConfiguration	sc_id, index_type, db_sc_id, buffer_size, flushing_unit_size, flushing_policy, log_size
eFINDConfiguration	sc_id, index_type, db_sc_id, buffer_size, read_buffer_perc, temporal_control_policy, read_temporal_control_perc, write_temporal_control_size, write_temporal_control_mindist, write_temporal_control_stride, timestamp_percentage, flushing_unit_size, flushing_policy, read_buffer_policy, log_size
OccupancyRate	or_id, min_fill_int_nodes, min_fill_leaf_nodes, max_fill_int_nodes, max_fill_leaf_nodes
IndexConfiguration	config_id, src_id, bc_id, sc_id, buf_id
SpatialIndex	idx_id, config_id, idx_name, idx_path, idx_creation, idx_last_mod
Execution^a^	pe_id, idx_id, execution_name, total_time, index_time, read_time, write_time, split_time, reads_num, writes_num, total_cpu_time, index_cpu_time, read_cpu_time, write_cpu_time, split_cpu_time,...
ReadWriteOrder	pe_id, rw_order, op_type, op_timestamp, page_id
FlashSimulatorStatistics	pe_id, read_count, write_count, erase_count, read_latency, write_latency, erase_latency
IndexSnapshot^a^	pe_id, height, num_int_nodes, num_leaf_nodes, num_entries_int_nodes, num_entries_leaf_nodes, avg_num_entries_pnode, avg_coverage_area_pnode,...
PrintIndex	pe_id, node_id, geom, elem_position, node_height

All attributes are fully described in the FESTIval's documentation at https://accarniel.github.io/FESTIval/.

a Attributes were suppressed.

There are two categories of data managed by FESTIval: (i) configuration of a spatial index, and (ii) storage of statistical data.

*Configuration of a spatial index*. It consists of four components. The first component is the *spatial dataset*, which is the source of spatial objects to be used by a spatial index. A spatial dataset is a PostgreSQL relational table that contains a column storing spatial objects. The needed information of spatial datasets is stored in the table Source. To insert a new spatial dataset in the FESTIval's data schema, we should provide its PostgreSQL schema name (*schema_name*), table name (*table_name*), column name that contains spatial objects (*column_name*), and the primary key of this table (*pk_name*). Hence, each tuple in Source represents a dataset that can be indexed. By using this strategy, users can use any spatial dataset in experiments. This fact contributes to providing a versatile platform to conduct empirical analyses.

The second component of a configuration refers to *basic parameters* that are employed by any spatial index. Basic parameters are stored in the table BasicConfiguration, which contains the storage system that stores the index (*ss_id*), the page size in bytes that an index page should have (*page_size*), the method of access to the storage device (*io_access*), and the library used in the refinement step in the spatial query processing (*refinement_type*). Currently, the attribute *io_access* is either the classical access to storage devices (i.e., using the *libio.h* in C) or the DIRECT I/O (i.e., using the *fcntl.h* in C) that allows us to bypass the caching system of reads and writes of the operating system; while the attribute *refinement_type* is either the use of the GEOS library or the use of PostGIS algorithms. As for the storage system, related data is stored in the table StorageSystem. This table contains the type of the storage device (*storage_system*) and its description (*description*). It can be HDDs, SSDs, or simulated flash memories using Flash-DBSim. For simulated flash memories, the primary key of the StorageSystem is linked to the table FlashDBSimConfiguration, which is an aggregated table of two needed information of the Flash-DBSim: (i) the flash device (table VirtualFlashDevice), and (ii) the flash translation layer (table FlashTranslationLayer). The attributes of these tables correspond to the same parameters required by Flash-DBSim, represented by the table FlashDBSimConfiguration, to simulate a flash memory as detailed in [24] and in the FESTIval's documentation.

The third component of a configuration refers to the *generic buffer management* of the spatial index. A generic buffer manager is a general-purpose method employed to reduce the number of accesses to the storage device; thus, any spatial index can employ a buffer manager. Parameters of the generic buffer manager are stored in the table BufferConfiguration. The attributes of this table consist of the size of the buffer in bytes (*buf_size*), and the type of the page replacement algorithm (*buf_type*). Currently, FESTIval provides support for the following buffer managers: LRU [26], LRU storing preferentially the highest nodes of the tree, called HLRU (as used in [18]), and the two versions of 2Q [27]. The management of generic buffers flushes modifications whenever their size is reached. The size of the buffer of a spatial index equal to 0 means that the spatial index has not a general buffer manager. This is the case if the spatial index has its own specialized buffer manager. For instance, flash-aware spatial indices (e.g., FAST-based and eFIND-based indices) have specialized buffer managers with specific parameter values (see below). Although it is possible to also employ generic buffer managers in flash-aware spatial indices, performance evaluations usually do not employ general buffer managers when analyzing the performance of flash-aware spatial indices [16], [17], [18], [19], [28].

Finally, the fourth component of a configuration refers to *specific parameters* that are used by an index. That is, each spatial index has its own set of parameters, and the table SpecializedConfiguration generalizes this concept by providing a unique identifier for this specific set of parameters. For instance, the R-tree permits to specify its split algorithm (*split_type*), which can be exponential, quadratic, and linear [3]. Other split algorithms are also conceivable, such as the Greene-split [29] and the AngTan-split [30]. This specific information is stored in the specialized table RTreeConfiguration and for each entry in this table, there is also an entry in the table SpecializedConfiguration that includes a short description (*description*). This strategy is similarly employed to store the specific parameters of other supported indices, that is, the R*-tree (table RStarTreeConfiguration), the Hilbert R-tree (table HilbertRTreeConfiguration), the FOR-tree (table FORTreeConfiguration), FAST-based indices (table FASTConfiguration), and eFIND-based indices (table eFINDConfiguration). The attributes of these tables are based on the corresponding parameters of their indices as specified in their original research papers. For FAST- and eFIND-based spatial indices, the use of the attribute *db_sc_id* that refers to the identifier of an entry of the SpecializedConfiguration allows us to combine the specific parameters of the underlying index pointed by this attribute and the specific parameters of FAST and eFIND.

In addition, we can vary the *occupancy rate* (table OccupancyRate) of index pages. This occupancy rate is informed by the attribute *or_id* that is present in the tables storing specific parameters of indices. We can specify the maximum capacity of leaf and internal nodes by using percentage values *max_fill_leaf_nodes* and *max_fill_int_nodes*, respectively. These percentage values specify how much space from the page size should be allocated to accommodate node entries. We can also specify the minimum capacity of leaf and internal nodes using respectively the attributes *min_fill_leaf_nodes* and *min_fill_int_nodes*, indicating the minimum occupancy rate considering the total available space (calculated from the maximum capacity of the nodes).

FESTIval provides an SQL script, named *festival-inserts.sql*, that contains SQL INSERT INTO statements for inserting default parameter values into all the aforementioned relational tables related to the configuration of spatial indices. As for the default spatial datasets, they can be downloaded at https://github.com/accarniel/FESTIval/wiki/ and a detailed specification of them is also given in [31]. Users are also able to insert new values to the aforementioned tables by executing SQL INSERT INTO statements. The informed values are checked by using triggers and SQL CHECK constraints in order to ensure the consistency of the parameters. For instance, it is impossible to insert a new R*-tree configuration that has a reinsertion type not defined in the original R*-tree paper.

The combination of the values of the attributes *src_id*, *bc_id*, *sc_id*, and *buf_id* of the tables Source, BasicConfiguration, SpecializedConfiguration, and BufferConfiguration, respectively, creates one spatial index configuration (table IndexConfiguration). Then, a spatial index (table SpatialIndex) consists of a configuration from the table IndexConfiguration (*config_id*) and other specific data, such as the name of the index (*idx_name*), the directory storing the index file (*idx_path*), the time of its creation (*idx_creation*), and the time of its last modification (*idx_last_mod*). Note that multiple spatial indices might have the same configurations; however, their states (i.e., content) might be different because of the executed operations. Only FESTIval insert entries into the tables IndexConfiguration and SpatialIndex, that is, users should not manually insert entries into these relational tables.

*Storage of statistical data*. FESTIval collects and stores two types of statistical data. The first type refers to *statistical data collected from the execution of index operations*. This data is maintained in the table Execution, which is a non-normalized table in order to avoid excessive joins when retrieving performance results. Each entry in this table means that at least one index operation like insertion, deletion, and query was performed. To identify the type of execution, the user can set a name (*execution_name*). This aspect is further discussed in the next sections of this article. Here, we only provide a general view of the main attributes of the table Execution: the total processing time of the index (*index_time*), the time spent to perform reads and writes (*read_time* and *write_time*, respectively), the processing time to execute splitting operations (*split_time*), the number of reads and writes (*reads_num* and *writes_num*, respectively), the CPU time of processing specific operations (e.g., *index_cpu_time*), and other attributes. Note that the total processing time of the index considers other times, such as the processing time of splits, reads, and writes. By collecting and storing detailed statistical data, FESTIval allows us to better analyze the composition of the total processing time (*total_time*). Further, FESTIval stores the order of reads and writes performed on the storage device (table ReadWriteOrder). To this end, a sequential identifier of the operation (*rw_order*), the type of operation (*op_type*, which can be either read or write), the moment that the operation was performed (*op_timestamp*), and the identifier of the index page (*page_id*) are stored. The order of reads and writes is optionally collected and is useful to discover data access patterns. Finally, if the executed workload employed a simulated flash memory, statistical data collected by Flash-DBSim are stored in the table FlashSimulatorStatistics. This data includes the number of read, writes, and erases actually performed on the simulated flash memory (*read_count*, *write_count*, and *erase_count*, respectively).

The second type of collected statistical data refers to *the structure of the spatial index*. This data is stored in the table IndexSnapshot. Each entry in this table allows us to analyze the structure of an index after executing operations that modify its structure, such as insertions and deletions. Here, we only provide a general view of the main attributes of the table IndexSnapshot: the height of the index (*height*), the number of internal and leaf nodes (*num_int_nodes* and *num_leaf_nodes*, respectively), the number of entries in internal and leaf nodes (*num_entries_int_nodes* and *num_entries_leaf_nodes*, respectively), summary data per node (e.g., the average number of entries – *avg_num_entries_pnode*), and other related attributes. Further, FESTIval provides the table PrintIndex that allows us to graphically visualize a spatial index by using a GIS, such as QGIS^4^ and ArcGIS.^5^ To this end, FESTIval stores data related to each node entry of the spatial index, such as position in the node (*elem_position*), its minimum bounding rectangle (MBR) (*geom*), height of the node (*node_height*), and the identifier of the node (*node_id*). This allows us to understand the structure of a spatial index for different purposes, such as educational.

#### The FESTIval's operations

FESTIval provides a set of SQL functions that allows users to create and execute workloads by using a common design. Each SQL function has the prefix *FT_* and calls one C function internally implemented in the FESTIval's internal library that is responsible for performing the desired processing. Hence, an index can be seen as an *abstract data type* [32] that has common parameters (i.e., its configuration) and a set of operations (i.e., SQL functions). The main advantage of this strategy is that complex implementations are hidden from users, who now can manage and test different indices under the same environment (i.e., using the same SQL functions).

There are three types of operations: (i) general operations, (ii) auxiliary operations, and (iii) atomic operations. They are described as follows.

*General operations*. They are responsible for handling index structures. Since FESTIval provides a common design, the same SQL function can be used for any type of index. We detail each general operation of FESTIval as follows:

1. boolean FT_CreateEmptySpatialIndex(integer index_type, text apath, integer src_id, integer bc_id, integer sc_id, integer buf_id);
2. boolean FT_Insert(text apath, integer pointer, geometry geom);
3. boolean FT_Delete(text apath, integer pointer, geometry geom);
4. boolean FT_Update(text apath, integer old_pointer, geometry old_geom, integer new_pointer, geometry new_geom);
5. setof query_result FT_QuerySpatialIndex(text apath, integer query_type, geometry search_obj, integer predicate, integer proc_option=1);
6. boolean FT_ApplyAllModificationsForFAI(text apath);
7. boolean FT_ApplyAllModificationsFromBuffer(text apath);

All these functions have a common parameter, *apath*, that indicates the absolute path of the index file. The first SQL function creates an empty spatial index (i.e., without any spatial objects) according to a set of parameters. It returns *true* if the index is successfully created, and *false* otherwise. The parameter *index_type* is an identifier that specifies the type of index to be created. Currently, FESTIval employs integer values from 1 to 10 to respectively represent the R-tree, the R*-tree, the Hilbert R-tree, the FAST R-tree, the FAST R*-tree, the FAST Hilbert R-tree, the FOR-tree, the eFIND R-tree, the eFIND R*-tree, and the eFIND Hilbert R-tree. The parameter *src_id* is a primary key value of the table Source that binds the spatial objects to the index. The parameters *bc_id*, *sc_id*, and *buf_id* specify the basic, specific, and buffer parameters of the spatial index by using the primary key values originated from the tables BasicConfiguration, SpecializedConfiguration, and BufferConfiguration, respectively. Since the specific parameters refer to only one type of index, FESTIval checks if the index to be constructed (i.e., the parameter *index_type*) is compatible with the values of *sc_id*. In summary, *FT_CreateEmptySpatialIndex* prepares all internal structures needed to handle a spatial index.

The three SQL functions *FT_Insert*, *FT_Delete*, and *FT_Update* execute operations that modify the index structure by respectively inserting, deleting, and updating spatial objects. They return *true* if the modification is successfully executed, and *false* otherwise. To insert and delete spatial objects, two additional parameters are needed: *pointer* and *geom*. The parameter *pointer* is the primary key value of the spatial object being inserted (or deleted), while the parameter *geom* is the geometry representing the spatial object. Spatial objects handled by FESTIval are PostGIS objects (i.e., *geometry* objects), guaranteeing a full integration of FESTIval with spatial applications that use PostGIS. To update a spatial object, *FT_Update* requires information about the spatial object being updated (parameters *old_pointer* and *old_geom*) to a new value (parameters *new_pointer* and *new_geom*). In general, an update is an atomic operation that sequentially deletes the old spatial object (and its pointer) and then inserts the new spatial object (and its pointer).

To apply the functions *FT_Insert*, *FT_Delete*, and *FT_Update*, it is needed to first create the index file (i.e., *apath*) by using the function *FT_CreateEmptySpatialIndex*. Further, since a spatial index is related to a specific dataset, it is important to apply the corresponding modification firstly in its dataset. For instance, add a new spatial object with its primary key value as a new tuple in the indexed dataset by using an SQL INSERT INTO statement before calling *FT_Insert*. FESTIval does not perform any changes in the dataset; thus, the user should perform modifications in the dataset as needed. The main advantage of this treatment is that we can isolate the time processing of a modification performed on the spatial index from the modification performed on the dataset.

The fifth SQL function executes spatial queries. It is a set-returning function of the PostgreSQL. It returns a set of *query_result* rows, formed by a primary key value (*id*) and a spatial object (*geom*) of the indexed dataset. The parameter *query_type* specifies the type of spatial query to be processed. There are many types of spatial queries proposed in the literature [1], [2]. FESTIval provides support for *spatial selections* (*query_type*=1), *range queries* (*query_type*=2), and *point queries* (*query_type*=3). Spatial selection is a general type of query that returns a set of spatial objects that satisfy some topological predicate (e.g., overlap, inside) for a given spatial object, called *search object*. Range query is similar to spatial selection but considering the search object as a rectangular-shaped object. Point query specializes spatial selection by allowing only the use of *intersects* as the topological predicate and points as search objects. The parameter *search_obj* is the search object, which is a PostGIS object. Some restrictions with respect to the geometric format of *search_obj* may be applicable. If *query_type* is equal to 2, the MBR of *search_obj* is considered. If *query_type* is equal to 3, *search_obj* must be a simple point object. The parameter *predicate* specifies the topological predicate [33] to be used in the spatial query. It can assume the following topological predicates: *intersects*, *overlap*, *disjoint*, *meet*, *inside*, *coveredBy*, *contains*, *covers*, and *equals* (they are integer values from 1 to 8, respectively). Finally, the last parameter *proc_option* refers to the type of the result of the spatial query, which is often executed by using two steps, filter and refinement [1]. If *proc_option* is equal to 1, its default value, *FT_QuerySpatialIndex* yields the final result of the query, that is, it executes the filter and refinement steps. In this case, it is important to maintain the spatial index compatible with the indexed dataset, as previously discussed. If *proc_option* is equal to 2, *FT_QuerySpatialIndex* returns the candidates of the query returned by the filter step only.

The last two SQL functions are responsible for executing a flushing operation that writes to the storage device all buffered index modifications. They return *true* if the flushing operation is successfully executed, and *false* otherwise. *FT_ApplyAllModificationsForFAI* flushes all buffered modifications stored in the specialized buffers of flash-aware spatial indices (i.e., eFIND-based indices), while *FT_ApplyAllModificationsFromBuffer* flushes all buffered modifications stored in the generic buffers (e.g., LRU, 2Q). These SQL functions write all modifications contained in the main memory, cleaning the corresponding buffer.

*Auxiliary operations*. They are designed for helping the process of creating workloads. They are mainly related to collecting and storing statistical data. We detail each auxiliary operation of FESTIval by providing its synopsis together with its short description as follows:

1. boolean FT_StartCollectStatistics(boolean rw=false);
2. boolean FT_CollectOrderOfReadWrite();
3. integer FT_StoreStatisticalData(text apath, integer statistic_option=1, integer loc_stat_data=1, text file=NULL);
4. boolean FT_StoreIndexSnapshot(text apath, integer execution_id, boolean print_index=false, integer loc_stat_data=1, text file=NULL);
5. boolean FT_SetExecutionName(text execution_name, integer loc_stat_data=1);

The functions returning Boolean values yield *true* if the processing is successfully performed, and *false* otherwise. The first auxiliary operation is the SQL function *FT_StartCollectStatistics*. After invoking this function, FESTIval starts to collect statistical data in the main memory. If the parameter *rw* is *false*, its default value, the order of reads and writes made on the storage device are not be collected. Otherwise, this order is collected, requiring extra computation. When an user performs the SQL function *FT_StartCollectStatistics*(*false*), and afterwards wants to collect the order of reads and writes, the user should call the SQL function *FT_CollectOrderOfReadWrite*(). This allows users to collect the order of reads and writes only for specific index operations since this collection is expensive; but it is important to understand access patterns.

Collected in-memory statistical data are only stored in the FESTIval's data schema after calling the SQL function *FT_StoreStatisticalData*, which returns an integer value that consists of the primary key value of the row inserted into the table Execution (i.e., the value of the column *pe_id*). The parameter *apath* is the absolute path of the index file and the parameter *statistic_option* refers to the type of statistical data that is stored. Independently of the value of *statistic_option*, FESTIval stores typical statistical data about the executed index operations (i.e., the attributes of the table Execution). If *statistic_option* is equal to 1, its default value, FESTIval inserts a new tuple in the table Execution only, without any other additional information. Optionally, FESTIval stores a new tuple in the table IndexSnapshot (*statistic_option*=2 or *statistic_option*=4), requiring the traversal of all index pages in order to collect statistical data related to the index structure. Further, FESTIval stores new tuples in the table PrintIndex (*statistic_option*=3 or *statistic_option*=4), also requiring the traversal of all index pages to visualize the index structure. The cost of traversing the tree is not take into account when collecting typical statistical data. Storing data in the tables IndexSnapshot and PrintIndex is particularly useful after the execution of operations that modify the index structure (e.g., insertions, deletions, and updates). As for the parameter *loc_stat_data*, it defines where (i.e., the location) the statistical data should be stored. If its value is equal to 1, its default value, the statistical data is stored directly in the FESTIval's data schema. If its value is equal to 2, the statistical data is stored in an SQL file that can be latter loaded into the FESTIval's data schema. In this case, the absolute path of this SQL file should be informed by using the parameter *file*. Particularly, setting the value 2 for *loc_stat_data* is useful to avoid reads and writes performed on the FESTIval's data schema during the execution of a workload. This aspect is important for SSDs because of the interference between reads and writes [13], [14]. Hence, the statistical data can be first stored in a file located in other storage device (e.g., an HDD). Finally, at any moment, the user is also able to collect and store statistical data related to the index structure by using the SQL function *FT_StoreIndexSnapshot*. It has almost the same parameters as the SQL function *FT_StoreStatisticalData*, expect for the parameters *execution_id* and *print_index*. The parameter *execution_id* is the primary key value of the table Execution that links the collected statistical data with an execution, while the parameter *print_index* indicates whether the structure of index should be collected and stored in the table PrintIndex or not.

The last auxiliary operation is the SQL function *FT_SetExecutionName*. Its main applicability is to set a name for the execution of a workload through the parameter *execution_name*. By creating workloads with this function, users are able to easily retrieve statistical data of executed workloads by issuing SQL queries on the table Execution. As previously described, the parameter *loc_stat_data* defines where the statistical data should be stored.

*Atomic operations*. They are combinations of some aforementioned functions and help the construction of workloads. An atomic operation is an SQL function that is executed as a unique and indivisible operation, following the principles of the atomicity of database systems [34]. That is, if any function inside an atomic operation fails, the entire atomic operation fails. In general, an atomic operation is formed by the following sequence of operations: (i) *FT_StartCollectStatistics*, (ii) the requested operation, and (iii) *FT_StoreStatisticalData*. The atomic operations of FESTIval start with *FT_A* and are specified as follows:

1. integer FT_AInsert(text apath, integer pointer, geometry geom, integer statistic_option=1, integer loc_stat_data=1, text file=NULL);
2. integer FT_ADelete(text apath, integer pointer, geometry geom, integer statistic_option=1, integer loc_stat_data=1, text file=NULL);
3. integer FT_AUpdate(text apath, integer old_pointer, geometry old_geom, integer new_pointer, geometry new_geom, integer statistic_option=1, integer loc_stat_data=1, text file=NULL);
4. setof query_result FT_AQuerySpatialIndex(text apath, integer query_type, geometry search_obj, integer predicate, integer proc_option=1, integer statistic_option=1, integer loc_stat_data=1, text file=NULL);

Algorithm 1 depicts the source code of the SQL function *FT_AInsert*, which illustrates a first usage of FESTIval's operations. The remaining atomic operations have very similar codifications. In fact, *FT_AInsert*, *FT_ADelete*, *FT_AUpdate*, and *FT_AQuerySpatialIndex* are atomic versions of the functions that respectively insert, delete, update, and query spatial objects from a spatial index. They combine the parameters of the SQL function responsible for executing the index operation and the parameters of *FT_StoreStatisticalData*. They assume that the order of reads and writes is not collected. Hence, collecting this kind of information requires the invocation of the SQL function *FT_CollectOrderOfReadWrite* before executing an atomic operation.

Algorithm 1 The source code of the SQL function *FT_AInsert*, an atomic operation of FESTIval

**Figure:**
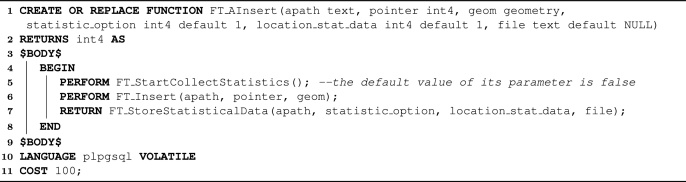

#### Creating and executing workloads

FESTIval provides a common design to create workloads. A workload consists of a sequence of index operations and can be created by using the SQL Procedural Language of the PostgreSQL (PL/pgSQL). Hence, users create workloads as user-defined functions in PL/pgSQL and execute them in SQL SELECT statements. Here, we illustrate two examples of workload and describe different scenarios of capturing performance results after executing a workload.

The first workload, depicted in Algorithm 2, executes a sequential insertion of spatial objects stored in a given dataset. That is, it constructs a spatial index by inserting spatial objects one-by-one. Due to the importance of this workload, FESTIval includes this function in its source code. The inputs of *FT_CreateSpatialIndex* are similar to those of the aforementioned SQL functions, except for the Boolean parameters *apply_fai* and *apply_stdbuffer* (line 1) that are employed to decide whether flushing operations should be performed in the end of the index creation. First, *FT_CreateSpatialIndex* extracts needed data about the dataset that is being indexed (line 10). This includes the names of its schema, table, column storing spatial objects, and primary key column. Then, the total number of rows of this table is retrieved (line 11) to identify how many spatial objects should be inserted into the spatial index. Afterwards, statistical data should be collected when executing the next index operations (line 12). The first index operation is the creation of an empty spatial index (line 13). Next, a sequence of insertions is made (lines 15–25). To better manage the main memory, the workload retrieves 100,000 spatial objects by time from the dataset (lines 16 and 17). The loop stops when all spatial objects are inserted into the spatial index (lines 19–21). In the sequence, the workload checks whether a flushing operation should be made in order to write all the remaining in-memory modifications after the insertions (lines 26–31). This includes the specialized in-memory buffer managers of flash-aware spatial indices (lines 26–28), and general buffer managers (lines 29–31). Finally, the workload stores statistical data related to the creation of the spatial index (line 32).

Algorithm 2 The workload written in PL/pgSQL for creating spatial indices

**Figure:**
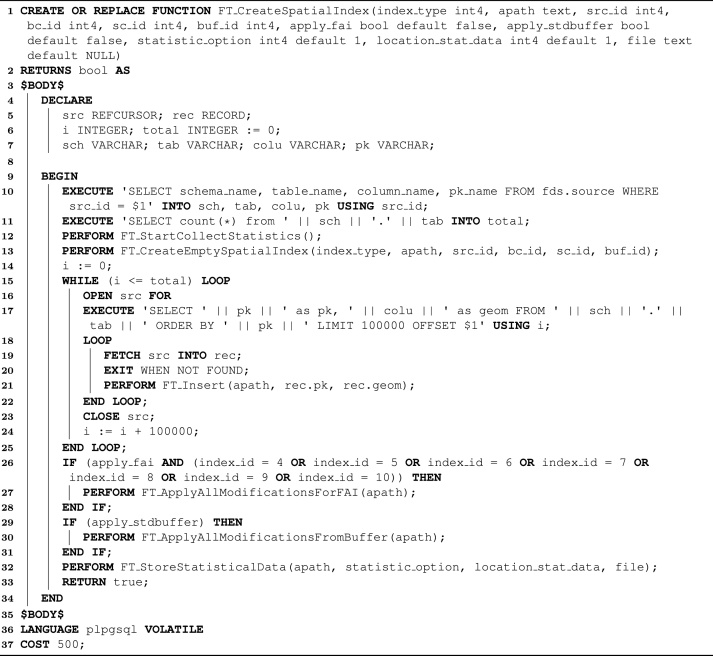

Before introducing the second workload, we show how the workload depicted in Algorithm 2 can be employed and how users can collect performance results. For this, we show a set of SQL statements that can be incrementally executed in order to reproduce our example.

In order to provide a name for the execution of *FT_CreateSpatialIndex*, we need to first execute an SQL SELECT statement that invokes *FT_SetExecutionName*, as shown below:

SELECT FT_SetExecutionName(‘Creating R-tree on brazil_points2017’);

Then, an index can be created by executing *FT_CreateSpatialIndex*. The next SQL SELECT statement creates an R-tree, called *linear_rtree* and stored in */opt/*, that indexes the spatial objects stored in the dataset identified by *src_id*=7 and with the parameter values *bc_id*=6, *sc_id*=18, and *buf_id*=4. These values are included in *festival-inserts.sql*, which can be loaded into the FESTIval's data schema after installing FESTIval. More precisely, this command builds an R-tree with page (node) size of 4 KB, employing the linear splitting algorithm, and a general LRU buffer of 512 KB. The indexed spatial objects are from the dataset named *brazil_points2017* [31]. Since this command does not change default values for arguments of *FT_CreateSpatialIndex*, it does not flush any modification remaining in the buffer after the insertions, and it stores statistical data only for the table Execution that is directly inserted into the FESTIval's data schema.

SELECT FT_CreateSpatialIndex(1, ‘/opt/linear_rtree’, 7, 6, 18, 4);

By issuing SQL SELECT statements, we are also able to retrieve and analyze performance results. For instance, the following command returns the required index time to build the previous R-tree (i.e., *linear_rtree*):

SELECT index_time
FROM fds.execution
WHERE execution_name = ‘Creating R-tree on brazil_points2017’;

Note the importance of setting a name for the execution, which can be used to retrieve performance results of executed workloads. The previous SQL SELECT statement could also include other columns containing statistical values. For instance, the next SQL SELECT statement yields the number of writes and reads required by the creation of the previous R-tree (i.e., *linear_rtree*).

SELECT reads_num, writes_num
FROM fds.execution
WHERE execution_name = ‘Creating R-tree on brazil_points2017’;

If *FT_CreateSpatialIndex* is executed multiple times, we are able to capture average statistical results from these executions. In this case, the index is built with different names but with the same configurations. Assuming that the previous R-tree is created multiple times (with different names, such as *linear_rtree2*, *linear_rtree3*, and so on), the following SQL SELECT statement returns the average index time and its standard deviation of these executions:

SELECT avg(index_time), stddev(index_time)
FROM fds.execution
WHERE execution_name = ‘Creating R-tree on brazil_points2017’;

Comparing performance results between two or more spatial indices is another scenario in which FESTIval helps users. For instance, consider the execution of the following two SQL SELECT statements. The first statement sets a new execution name since we are dealing with a different spatial index, the R*-tree. The second statement creates an R*-tree, named *rstartree* and stored in */opt/*, using the same node size of 4 KB (i.e., *bc_id*=6) and general LRU buffer of 512 KB (i.e., *buf_id*=4) that indexes the same dataset as the previous R-tree (i.e., *src_id*=7). The specific parameter *sc_id*=60 refers to the configuration of the R*-tree that specifies the reinsertion of 30% of entries according to the CLOSE REINSERT policy. Further, this command does not change default values for arguments of *FT_CreateSpatialIndex*.

SELECT FT_SetExecutionName(‘Creating R*-tree on brazil_points2017’);
SELECT FT_CreateSpatialIndex(2, ‘/opt/rstartree’, 7, 6, 60, 4);

Considering that the previous R-tree and R*-tree have been built the same number of times and with different names, we are able to compare their average time of creation as follows:

SELECT execution_name, avg(index_time), stddev(index_time)
FROM fds.execution
WHERE execution_name IN (‘Creating R-tree on brazil_points2017’,
‘Creating R*-tree on brazil_points2017’);

Another example of execution of *FT_CreateSpatialIndex* is to vary its parameter values in order to collect statistical data related to the index structure, as shown in the next two SQL SELECT statements. The first one denominates the corresponding execution name. The second one creates another R-tree, called *linear_rtree_comp* and stored in */opt/*, with the same parameter values of the previous R-tree (i.e., *linear_rtree*); but collecting statistical data related to its structure, which is useful to analyze the spatial organization of the index. In addition, FESTIval also stores the nodes of the built index, which is useful to visualize the index by using specialized programs like QGIS.

**Fig. 3:**
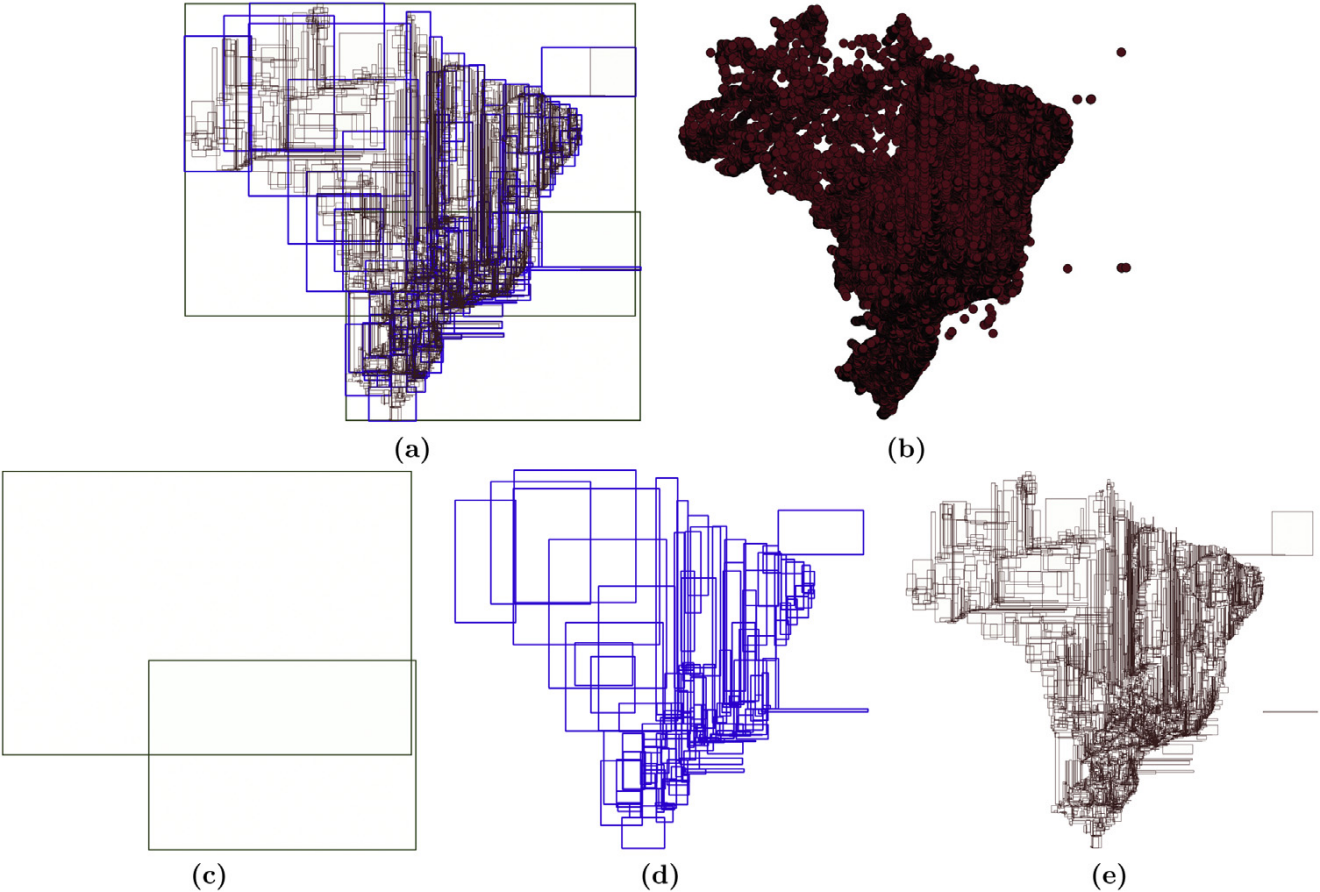
Visualization of the MBRs of node entries of an R-tree. This R-tree (a) is built over the *brazil_points2017* (b) and has height equal to 3. The entries of each level, from the highest to the lowest level, are shown in (c), (d), and (e), respectively.

SELECT FT_SetExecutionName(‘Creating R-tree on brazil_points2017’);
SELECT FT_CreateSpatialIndex(1, ‘/opt/linear_rtree_comp’, 7, 6, 18, 4, false, false, 4);

The next SQL SELECT statement shows an example of a query that returns the height, the number of internal and leaf nodes, and the average number of entries per node of the previously built R-tree (considering that only the aforementioned SQL statements were executed):

SELECT height, num_internal_nodes, num_leaf_nodes, avg_num_entries_pnode
FROM fds.execution e, fds.indexsnapshot is
WHERE e.pe_id = is.pe_id AND execution_name = ‘Creating R-tree on brazil_points2017’;

Further, the user is also able to visualize this index by retrieving rows from the PrintIndex. Every row in this table represents an entry of an index page, which has at least a pointer, height, and a geometry object representing its MBR. Fig. 3 depicts the structure of the built R-tree (i.e., *linear_rtree_comp*) by using the QGIS. Different layers of geometries are employed to see the MBRs of each level of the tree (Fig. 3c–e). This visualization is particularly useful to graphically represent indices, such as for educational purposes.

Algorithm 3 The workload written in PL/pgSQL for executing spatial queries

**Figure:**
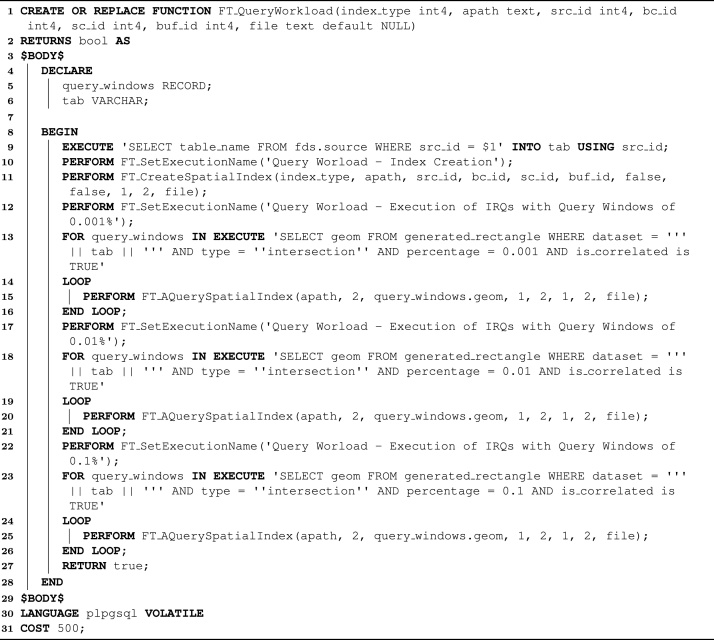

Algorithm 3 depicts another workload, named *FT_QueryWorkload*. This workload has been used to understand the impact of SSDs on the spatial indexing context [10], [11], [28], [35], [36] and to measure the performance gains of eFIND [18], [19]. Its main goal is to build an index and to execute intersection range queries (IRQs). The employed query windows (stored in the relational table called *generated_rectangle*) are correlated to the indexed dataset [31] and are available at https://github.com/accarniel/FESTIval/wiki/. All inputs of this workload have the same meaning of the common inputs of the workload depicted in Algorithm 2. *FT_QueryWorkload* first gets the name of the dataset to be indexed in this workload (line 9). Then, the workload sets the name of the execution (line 10) to identify that the next operation is the index construction (line 11). After building the index, three different sets of IRQs are processed (lines 12–16, lines 17–21, and lines 22–26). To this end, three different sets of query windows are employed. Each set has 100 query windows with specific sizes of the area of the total extent of Brazil. These sizes are 0.001%, 0.01%, and 0.1%, respectively. Considering that the selectivity of a spatial query is the ratio of the number of returned objects and the total objects, these sets of query windows form spatial queries with low, medium, and high selectivity, respectively. Each execution of a spatial query is performed by the atomic operation *FT_AQuerySpatialIndex*. Finally, all statistical data is stored in a file located in the HDD (parameter *file* in lines 11, 15, 20, and 25) since this workload handles the spatial index file stored in an SSD. The use of this workload to validate a flash-aware spatial index is further discussed in the next section.

## Method validation: employing FESTIval to measure the efficiency of eFIND

In this section, we show how *FT_QueryWorkload* (Algorithm 3) is employed to validate eFIND and how the statistical results can be extracted to measure its performance gains. eFIND [18], [19] is a generic approach that transforms a disk-based spatial index (e.g., the R-tree) into an efficient flash-aware spatial index (e.g., eFIND R-tree). The examples described in this section details a part of the experimental evaluation conducted by us in [19]. These experiments compare eFIND against FAST, which is the closest competitor to eFIND among existing approaches to implementing flash-aware spatial indices. eFIND and FAST are employed to port the traditional R-tree to SSDs, forming the following configurations: the *eFIND R-tree* and the *FAST R-tree*, respectively. The used spatial dataset (i.e., *src_id*) is the *brazil_buildings2017* [31], containing 1,485,866 regions that represent the buildings of Brazil. Both configurations employed an in-memory buffer of 512 KB, log size of 10 MB, and the flushing unit size equal to 5. The best parameter values were applied for the remaining specific parameters. This means that we did not vary specific parameter values for each configuration (i.e., *sc_id=50031* for the eFIND R-tree and *sc_id=1323* for the FAST R-tree). On the other hand, basic parameter values (i.e., *bc_id*) are varied to evaluate the eFIND R-tree and the FAST R-tree under page sizes from 2 KB to 32 KB. A generic buffer was not employed in the experiments (i.e., *buf_id=1*). We conducted the experiments on a Kingston SSD V300 of 480 GB.

The next SQL SELECT statements execute the workload depicted in Algorithm 3 to evaluate the performance of the eFIND R-tree and the FAST R-tree, respectively, using the page size equal to 4 KB (i.e., *bc_id=53*). The files *efind_results.sql* and *fast_results.sql*, maintained in an HDD, are employed to store statistical data for the eFIND R-tree and the FAST R-tree, respectively:

SELECT FT_QueryWorkload(8, ‘/opt/efind_rtree1’, 5, 53, 50031, 1, ‘/HDD/efind_results.sql’);
SELECT FT_QueryWorkload(4, ‘/opt/fast_rtree1’, 5, 53, 1323, 1, ‘/HDD/fast_results.sql’);

Similar SQL SELECT statements are issued to evaluate the eFIND R-tree and the FAST R-tree for different page sizes. Each SQL SELECT statement is executed 5 times, varying the name of each index file, in order to calculate the average index time of the index construction and the execution of the IRQs. The cache of the PostgreSQL and the operating system is cleaned between the executions.

As previously discussed, collecting statistical data requires the execution of SQL SELECT statements on the FESTIval's data schema. For instance, the next query returns the average index time and its standard deviation to construct an eFIND R-tree for each employed page size:

SELECT b.page_size, avg(e.index_time), stddev(e.index_time)
FROM fds.basicconfiguration b, fds.specializedconfiguration sc,
fds.indexconfiguration ic, fds.spatialindex si, fds.execution e
WHERE b.bc_id = ic.bc_id AND sc.sc_id = ic.sc_id AND
ic.config_id = si.config_id AND si.idx_id = e.idx_id AND
ic.sc_id = 50031 AND execution_name = ‘Query Worload - Index Creation’
GROUP BY b.page_size
ORDER BY b.page_size;

The same structure of query can be also used for extracting performance results of the FAST R-tree.

In our analyses, we collected the average of the total elapsed time required to execute each set of 100 IRQs. The following SQL SELECT statement is performed when collecting results for the query windows with 0.001% of the area of Brazil:

SELECT t.page_size, avg(t.s), stddev(t.s)
FROM (
SELECT si.idx_name, b.page_size, sum(e.index_time) as s
FROM fds.basicconfiguration b, fds.specializedconfiguration sc,
fds.indexconfiguration ic, fds.spatialindex si, fds.execution e
WHERE b.bc_id = ic.bc_id AND sc.sc_id = ic.sc_id AND
ic.config_id = si.config_id AND si.idx_id = e.idx_id AND ic.sc_id = 50031 AND
execution_name = ‘Query Worload - Execution of IRQs with Query Windows of 0.001GROUP BY si.idx_name, b.page_size
) as t
GROUP BY t.page_size
ORDER BY t.page_size;

The subquery returns the sum of the index time required to process the 100 IRQs with query windows of 0.001% for each built index and page size. Note that we have 5 spatial indices with the same configurations created by the multiple executions of *FT_QueryWorkload*; each spatial index has a different name stored in *idx_name*. Then, the outer query returns the average and the standard deviation of the total elapsed time for each page size. A similar approach is used to extract the performance results of the FAST R-tree.

**Fig. 4:**
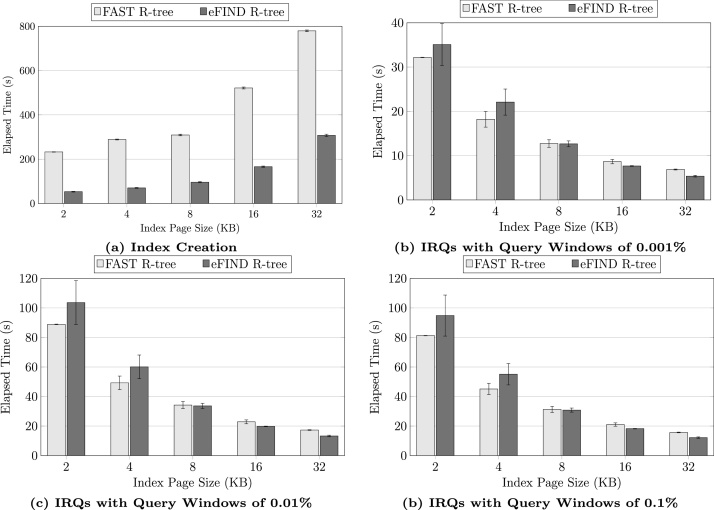
FESTIval was very useful to measure the performance gains of the eFIND R-tree, which reduced the time spent when building spatial indices (a) and when processing IRQs (b, c, and d).

By using the returned results of the SQL SELECT statements, we can employ data analytics tools (Fig. 1) to analyze the performance results. For instance, we can visualize the results by using bar graphs, where the *x*-axis is the first returned column (i.e., *page_size*) and the *y*-axis is the average time with the errors bar for the standard deviation, as shown in Fig. 4. In our experiments, the eFIND R-tree overcame the FAST R-tree when building indices in all employed page sizes. Its performance gains were very expressive, ranging from 60% to 77% for the Kingston SSD (Fig. 4a). As for the query processing (Fig. 4b–d), the eFIND R-tree provided the best performance only for larger pages, showing gains of 22% and 23% for the index pages of 16 KB and 32 KB, respectively. The efficiency of eFIND comes from the use of a set of design goals specified to fully exploit SSD performance [18].

## Conclusions and future work

In this article, we propose FESTIval, a versatile method for conducting experimental evaluations of spatial indices under the same environment. FESTIval is implemented as a PostgreSQL extension and includes the following advantages: (i) the support for disk-based and flash-aware spatial indices that can assume different configurations by setting their corresponding parameter values, (ii) the definition of user-defined workloads by using FESTIval's SQL functions, (iii) the use of any spatial dataset when executing workloads, (iv) the collection of different types of statistical data that are stored in the FESTIval's data schema, and (v) the reproduction of executed experiments.

The positive characteristics of FESTIval allow its use in distinct experimental evaluations, such as experiments for analyzing the impact of SSDs in the spatial indexing context [10], [11], and experiments for validating new proposals of spatial indexing on SSDs (e.g., eFIND-based indices [18], [19]). Moreover, external data analytics tools can access the FESTIval's data schema to generate different types of graphics, to plot maps, and to process statistical data in mathematical models. These aspects are very important when benchmarking spatial indexing structures in spatial database systems and GIS.

Future work will mainly deal with two topics. The first topic relates to the continuous development of FESTIval by including the support for other spatial indexing structures. We plan to provide support for the xBR^+^-tree [37] since this spatial index has been ported to SSDs using FAST and eFIND [38]. As a result, we also plan to include the FAST xBR^+^-tree and the eFIND xBR^+^-tree in FESTIval. The second topic consists of creating a systematic model that recommends the best spatial indices to be employed on SSDs according to a given context (e.g., the type of workload employed in the spatial application). The idea is to integrate this model into FESTIval as SQL functions.

## Footnotes

1 For more information about writing PostgreSQL extensions, please access related PostgreSQL documentation at https://www.postgresql.org/docs/10/static/extend-extensions.html.

2 https://postgis.net/.

3 http://trac.osgeo.org/geos.

4 https://qgis.org/.

5 https://www.arcgis.com/index.html.
