# Supplementary material for: FESTIval: A versatile framework for conducting experimental evaluations of spatial indices
Source: MethodsX. 2019 Oct 16;7:100695. doi: 10.1016/j.mex.2019.10.006 (PMC6994642; doi:10.1016/j.mex.2019.10.006)
Supplement: Supplementary file 1 [file mmc1.pdf]

Supplementary material and/or Additional information

Installation

FESTIval has the following dependencies: PostgreSQL 9.5 (or later), and PostGIS 2.2.1 (or later) with its dependencies. Further, a main dependency of PostGIS and, consequently of FESTIval, is the GEOS library. We strongly recommend the most recent version of GEOS for both systems. After configuring the Makefile of FESTIval, the user can install it by issuing the following commands in the terminal:

```
sudo make all
sudo make install
```

Once installed, FESTIval should be enabled in a PostgreSQL database for its usage. To this end, the following SQL CREATE EXTENSION statement should be issued:

```
CREATE EXTENSION festival;
```

The FESTIval's data schema is automatically created by FESTIval when it is enabled in a PostgreSQL database. The default values of the FESTIval's data schema can be loaded by executing the following command:

```
psql -U user -d database -vfestivaldir=/f_path/ -f /f_path/festival-inserts.sql
```

where *user* is the user name of the PostgreSQL, *database* is the database name in which FESTIval is enabled, and */f_path/* is the full path of the FESTIval's source code.

Previous version of FESTIval

The first version of FESTIval was described in [24]. The version introduced in this article greatly extends the previous version of FESTIval as follows. First, we extend FESTIval to provide support for other indexing structures, such as the Hilbert R-tree and flash-aware spatial indices. Second, FESTIval now also provides support for generic buffers, such as the LRU and the 2Q. Third, a common design was introduced to provide the creation of user-defined workloads. Fourth, spatial indices can be also evaluated on emulated flash memories. Finally, the current version of FESTIval also allows to manage refined statistical data, such as the order of read and writes.

We are constantly improving the source code of FESTIval. These extensions allow us to continuously use FESTIval in research papers, such as in [10, 11, 18, 19, 29, 36, 37].

Adding New Spatial Indexing Structures to FESTIval

FESTIval is designed to be an extensible method, allowing not only the specification of user-defined workloads but also the incorporation of new spatial indexing structures. This is possible because FESTIval represents a spatial index by using underlying concepts of *abstract data types*. That is, a spatial index is defined considering general and specific parameters and a set of operations. While the parameters of the spatial index are defined as a relational table in the FESTIval's data schema, the operations of the spatial index are internally implemented as SQL functions that call multiple dispatching functions of the C language. In this section we provide the key ideas for adding new spatial indexing structures to FESTIval. More details are given in the FESTIval's documentation.

The incorporation of a new spatial index into FESTIval consists of two steps as follows.

Incorporation of a new relational table into the FESTIval's data schema. The specific parameters of the new index should be modeled as attributes of a new relational table. This new table should be generalized by the SpecializedConfiguration (Figure 2). That is, for each entry of the new table, there is also a corresponding row in the SpecializedConfiguration indicating its unique identifier and a general description. This shows that the FESTIval's data schema is extensible. After creating and incorporating this new table, it is also needed to develop functions in the FESTIval's source code in order to access rows from this new table. For this, the current code of FESTIval that extracts the specific parameters of the supported spatial indices (e.g., the R-tree) should be used as a basis.

Incorporation of the implementation of the new spatial index into the FESTIval's source code. Since FESTIval is implemented in C, the new spatial index should be implemented in the C language or include a C interface. FESTIval represents a spatial index as a C structure called *SpatialIndex*. This structure is handled by the FESTIval's internal library, such as the implementation of the C functions responsible for implementing the FESTIval's general operations (e.g., *FT_Insert*). Hence, each supported spatial index is generalized by *SpatialIndex*. Its first element is another structure containing a set of function pointers providing the signatures of the index operations that should be implemented by every spatial index. For instance, they specify what are the inputs and returning values of basic index operations like insertion, update, deletion, and query. Based on that, the new spatial index should be represented as an instance of *SpatialIndex* that provides the corresponding function pointers to each element of the first internal structure of *SpatialIndex*. Other elements of *SpatialIndex* consist of additional structures storing data from the tables Source, BasicConfiguration, and BufferConfiguration; these structures should also be set accordingly when creating an instance of *SpatialIndex*.

As for the collection of statistical data, some of them are collected independently from the spatial index. For instance, the time required by the spatial index to process an operation. But this is not the case for the statistical data that depends on the implementation of the spatial index. For instance, the required time to execute a split operation. For these cases, the global variables of the FESTIval's library should be used, as detailed in the FESTIval's documentation.
